# β-Carotene: a natural osteogen to fabricate osteoinductive electrospun scaffolds†

**DOI:** 10.1039/c7ra13237a

**Published:** 2018-03-12

**Authors:** Atiyeh Dabouian, Hadi Bakhshi, Shiva Irani, Mohamad Pezeshki-Modaress

**Affiliations:** Department of Biology, School of Basic Sciences, Science and Research Branch, Islamic Azad University 1477893855 Tehran Iran; Macromolecular Chemistry II, University of Bayreuth, Universitätsstraße 30 95440 Bayreuth Germany; Tissue Engineering and Regenerative Medicine Institute, Tehran Central Branch, Islamic Azad University Tehran Iran hadi.bakhshi@uni-bayreuth.de s.irani@srbiau.ac.ir

## Abstract

β-Carotene (βC) as a natural osteogenic material was incorporated in PCL electrospun mats to fabricate scaffolds for bone tissue engineering. These scaffolds successfully supported the attachment and proliferation of mesenchymal stem cells (MSCs). Seeded scaffolds were calcinated during 21 days of cell culture in a non-differential medium, which showed the osteodifferentiation of MSCs. Expression of *RUNX2*, SOX9, and *osteonectin* proved the osteoinductive effect of incorporated β-carotene on the differentiation of MSCs to osteoblasts without using any external osteogenic differential agent. However, the cells did not pass the early phase of osteogenesis and were still osteochondro-progenitor after 21 days of incubation. Thus, the fabricated fibrous scaffolds are potential candidates for direct bone tissue engineering.

## Abstract 2

Electrospun PCL scaffolds containing β-carotene as a natural osteogenic material can differentiate MSCs to osteoblasts without using external differential agents.

## Graphical abstract

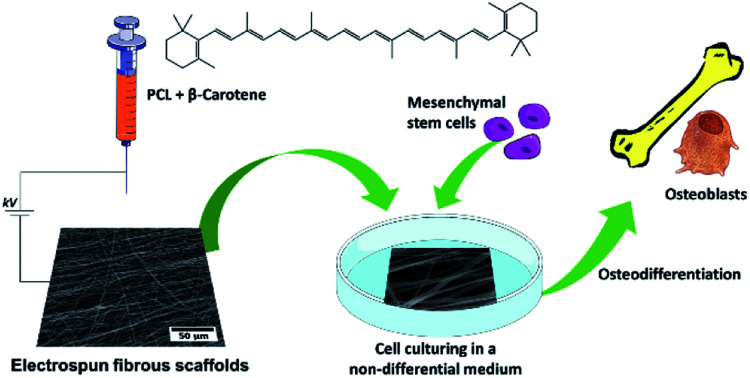

## Introduction

Bone fractures caused by osteoporosis affect about one-third of women and one-fifth of men aged over 50 years in the world^1^ and are the main reason for disability and suffering in the elderly population. Women, particularly postmenopausal women, are extremely vulnerable to osteoporosis.^2^ Despite the healability of bone tissue, restoration of some fractures and damage through normal physiological processes is very slow, particularly when the defect size is greater than the healing capacity of bone tissue.^3^ Thus, bone tissue engineering has attracted increasing interest for constructing biological substitutes that allow growth and proliferation of host cells in injured and diseased bone to restore and maintain normal function.^4–7^

Mesenchymal stem cells (MSCs), as a main source of adult stem cells, have found numerous applications in tissue engineering and cell therapy due to their ability to self-reproduce and differentiate into a range of cells and tissues such as bone, cartilage, muscle, tendon, ligament, and fat.^8–11^ MSCs have been the focus of recent studies in bone tissue engineering, in which they are seeded in a scaffold and induced to generate new osteogenic cells *via* osteoinductive cues.^12–14^ Appropriate bioactive cues encapsulated within the scaffolds can not only regulate cell adhesion and proliferation, but also enhance osteogenic differentiation. For example, incorporation of hydroxyapatite (HA) into the formulations of scaffolds enhanced the osteoinductivity^15,16^ since bone is primarily composed of HA^17^. Furthermore, encapsulation of drugs such as dexamethasone,^18,19^ simvastatin,^20^ bone morphogenetic proteins^21^ and ascorbate-2-phosphate^19^ within scaffolds and their sustained release improved the osteogenic differentiation of seeded MSCs.

**Fig. 1:**
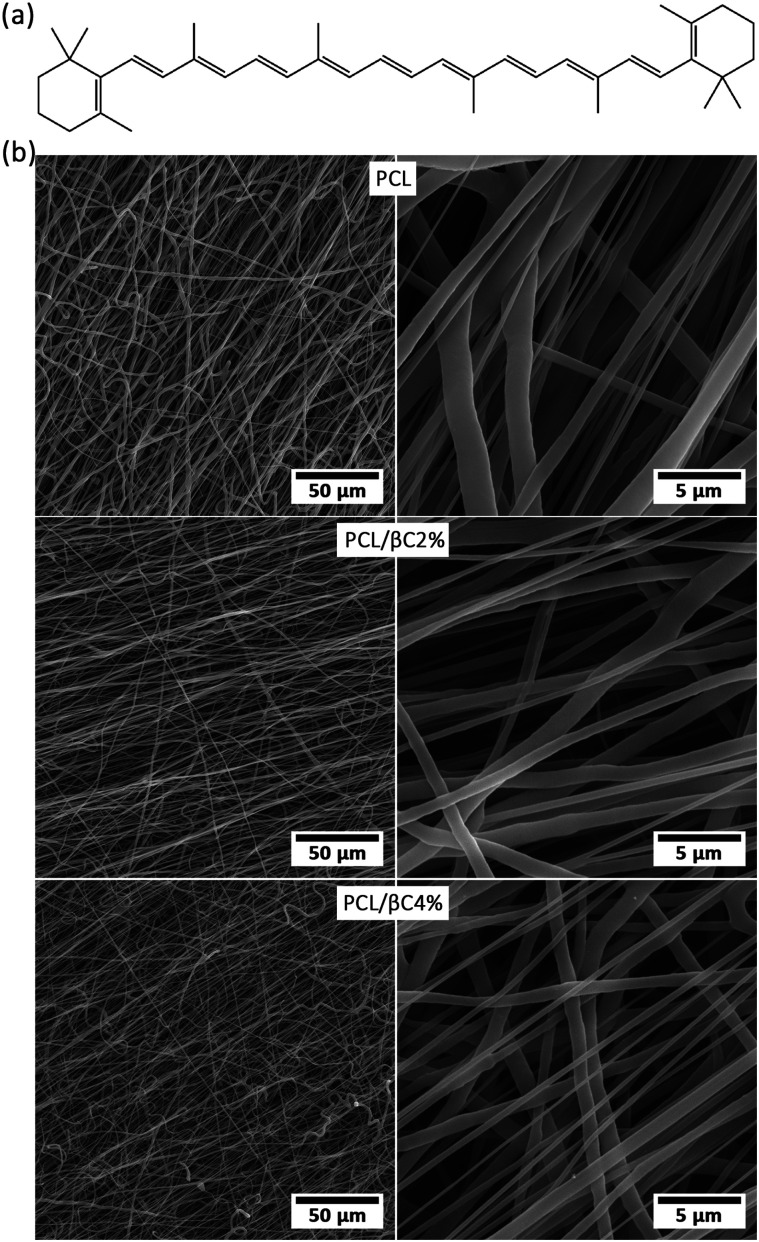
(a) The molecular structure of β-carotene. (b) SEM images of the fabricated random fibrous mats.

β-Carotene (βC) is the most abundant precursor of vitamin A in the human diet and found in dark-green and orange fruits and vegetables.^22,23^ It is a natural bioactive component with electrical activity^24,25^ due to its conjugated double bonds (Fig. 1a). It can reduce bone loss in elder adults.^26,27^ The ability of β-carotene to induce osteogenic differentiation of stem cells has been reported.^28–30^ Although there are some recent articles about encapsulation of β-carotene within electrospun fibers,^31–33^ based on our literature survey, no research has been reported on the incorporation of β-carotene within electrospun scaffolds for tissue engineering applications.

This research aims to fabricate PCL fibrous mats containing β-carotene as bone tissue engineering scaffolds with the ability to self-differentiate MSCs to osteoblasts without using any osteogenic differential agent. Polycaprolactone (PCL) was chosen as a polymer with high mechanical strength, lack of toxicity and low cost^34–36^ for fabricating scaffolds *via* an electrospinning process. First, the morphology and biocompatibility of the mats for bone tissue engineering were evaluated and then, their performance in simulating the appropriate environment for the differentiation of MSCs to osteoblasts was studied.

## Experimental

All experimental details, including materials, electrospinning process, instruments, and biological assays, are provided in the electronic supplementary information (ESI†).

## Results and discussion

### Fabrication of scaffolds

Electrospinning is a proficient technique for the fabrication of a porous substrate with high surface area, similar to extracellular matrix (ECM); such a porous substrate is suitable for cell attachment and proliferation.^18,37^ PCL fibrous mats containing β-carotene (0, 2 and 4 wt%, called PCL, PCL/βC2% and PCL/βC4%, respectively) were prepared *via* electrospinning process. SEM images (Fig. 1b) show that the fabricated mats consisted of randomly oriented fibers with an average diameter of 1.64 ± 1.42, 2.52 ± 1.49 and 1.87 ± 0.98 μm. The pore size of mats was 6.45, 5.15 and 2.34 μm, respectively. The porosity of the mats was 49.3, 52.8 and 48.2%. The mats contained large interconnected cavities, which are well-suited to provide the appropriate biological conditions for guiding MSCs to osteoblasts.

The chemical structure of the electrospun mats was investigated by FTIR spectroscopy (Fig. S1–S3 in ESI†). The FTIR spectra of all PCL mats were the same as that of virgin PCL, which indicated that no degradation or decomposition occurred during the electrospinning process. Due to the low content of β-carotene, no corresponding peak was observed in the FTIR spectra of the mats.

### Biocompatibility

**Fig. 2:**
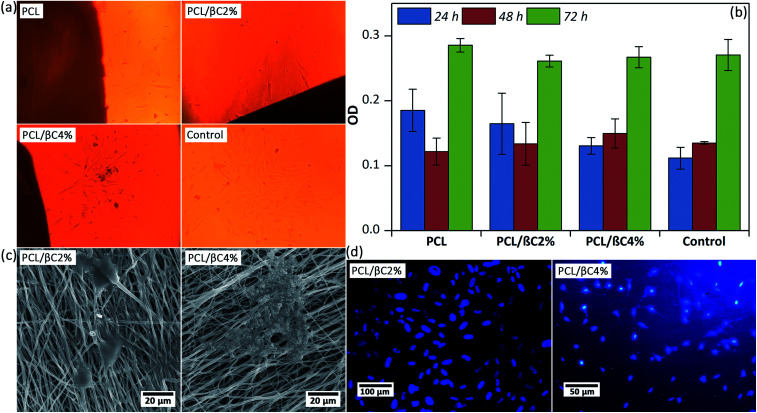
(a) Optical microscopic images of MSCs (10^4^ cells per well) on the scaffolds (0.5 × 0.5 cm^2^) after 24 hours. Tissue culture plate was used as the control. The magnification is 100. (b) The viability of MSCs on the scaffolds up to 76 hours obtained by MTT assay. (c) SEM images of the MSCs attached on the scaffolds after 48 hours. (d) DAPI staining images of the cell nucleus on the scaffolds after 48 hours.

Scaffolds designed for tissue engineering should be biocompatible, so that cells can attach to these scaffolds and proliferate on them. Thus, the viability of MSCs (10^4^ cell per well) on the scaffolds (0.5 × 0.5 cm^2^) after 24, 48, and 72 hours was evaluated. Optical microscope images (Fig. 2a) display that spindle-shaped MSCs have proliferated and moved toward the scaffold. The MTT results (Fig. 2b) indicate no cytotoxicity effects for the scaffolds up to 72 hours; the cell viability of MSCs considerably improved (*p* < 0.001) on the third day. Incorporation of β-carotene within mats did not significantly change (*p* > 0.05) the viability of seeded MSCs. Moreover, the cell proliferation on the scaffolds was similar to that on the tissue culture plate used as the control.

A good attachment of MSCs on the scaffold after 48 hours was observed in SEM images (Fig. 2c), in which well-developed cell–cell and cell–matrix interactions are observed. DAPI assay was used after 48 hours to evaluate the adhesion of seeded MSCs with healthy nuclei. These results confirmed that the fabricated scaffolds are biocompatible and can support the attachment and proliferation of MSCs.

### Osteoblast differentiation

**Fig. 3:**
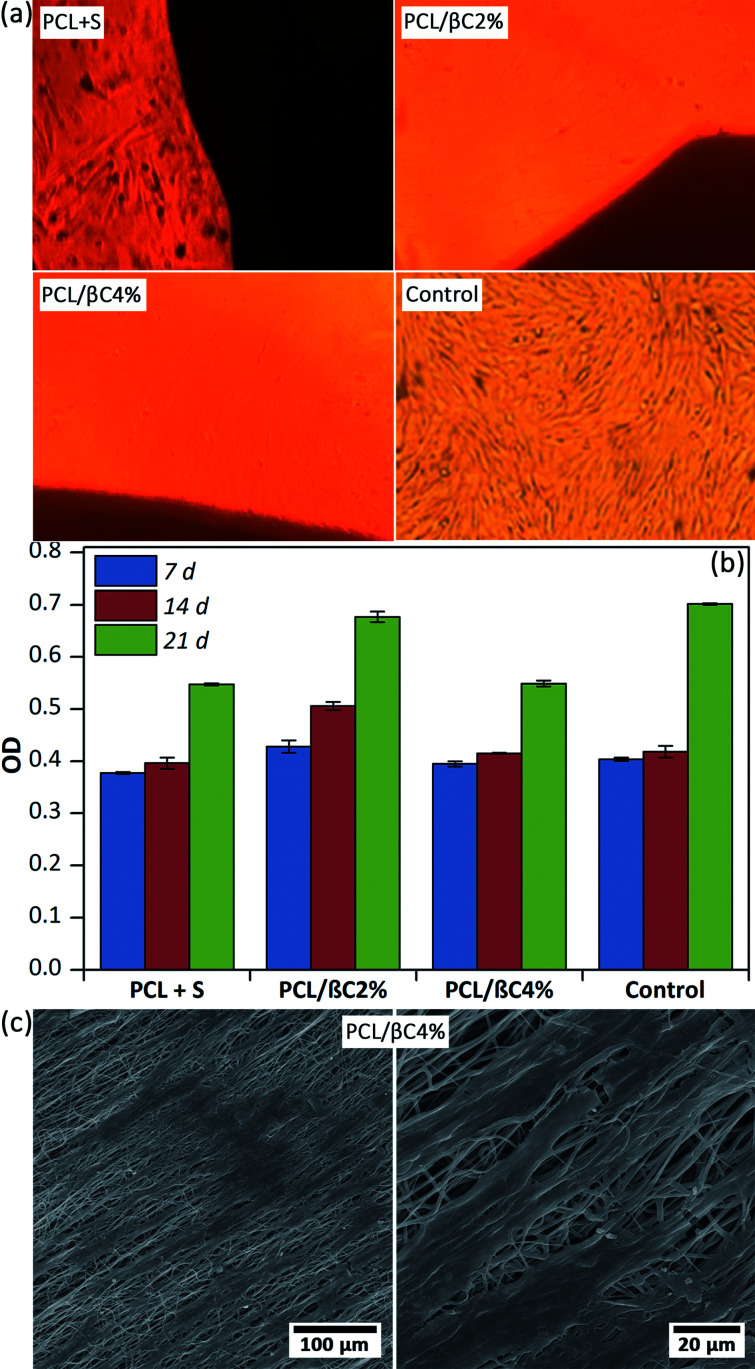
(a) Optical microscopic images of differentiated cells (10^4^ cell per well) on the scaffolds (0.5 × 0.5 cm^2^) after 21 days. Tissue culture plate was used as the control. S was l-ascorbic acid (10 mM) and dexamethasone (1 mM). The magnification is 100. (b) The viability of the cells on the scaffolds up to 21 days obtained by MTT assay. (c) SEM images of the cells on PCL/βC4% scaffold after 14 days.

Recent reports demonstrated the osteogenic effect of retinoic acid^38^ and β-carotene^2,28–30^ on cells. Nishide *et al.*^28^ studied the role of carotenoids in suppressing osteoclastogenesis. They activate cell signaling *via* binding to nuclear retinoic acid receptors (RARs). RARs attach to DNA as heterodimers with the retinoid X receptors (RXRs) and consequently modulate retinoic acid-responsive target genes.^28,39–41^ Wang *et al.*^2^ reported that β-carotene exerted an inhibitory effect on osteoclastogenesis and resorption pit formation through the nuclear factor-kappa B (NF-κB) pathway. To study the osteoinductive effect of β-carotene-containing mats on the differentiation of MSCs, scaffolds (PCL/βC2% and PCL/βC4%, 0.5 × 0.5 cm^2^) were seeded with cells (10^4^ cell per well) and kept in DMEM with 10% FBS as a non-differential medium for up to 21 days. For comparison, l-ascorbic acid (10 mM) and dexamethasone (1 mM) as external osteogenic differential agents^15^ were added to the cell culture medium used for the pure PCL scaffold. Optical microscopy images (Fig. 3a) shows the morphology change in osteodifferentiated cells on PCL/βC2% and PCL/βC4% scaffolds after 21 days as compared to that on the tissue culture plate used as the control. A similar change was observed for cells on pure PCL scaffold cultured in the presence of external differential agents.

The viability of cells on scaffolds during 21 days culture was evaluated by MTT assay (Fig. 3b). The number of cells on the scaffolds increased for up to 21 days, confirming their metabolic activity during this period. The viability of cells on PCL/βC4% scaffold significantly decreased during the third week (*p* < 0.001) as compared to that on PCL/βC2% scaffold and the control; this can be attributed to the differentiation of MSCs to osteoblasts. SEM images (Fig. 3c) show that the morphology of seeded MSCs on PCL/βC4% completely changed to osteoblast after 14 days.

**Fig. 4:**
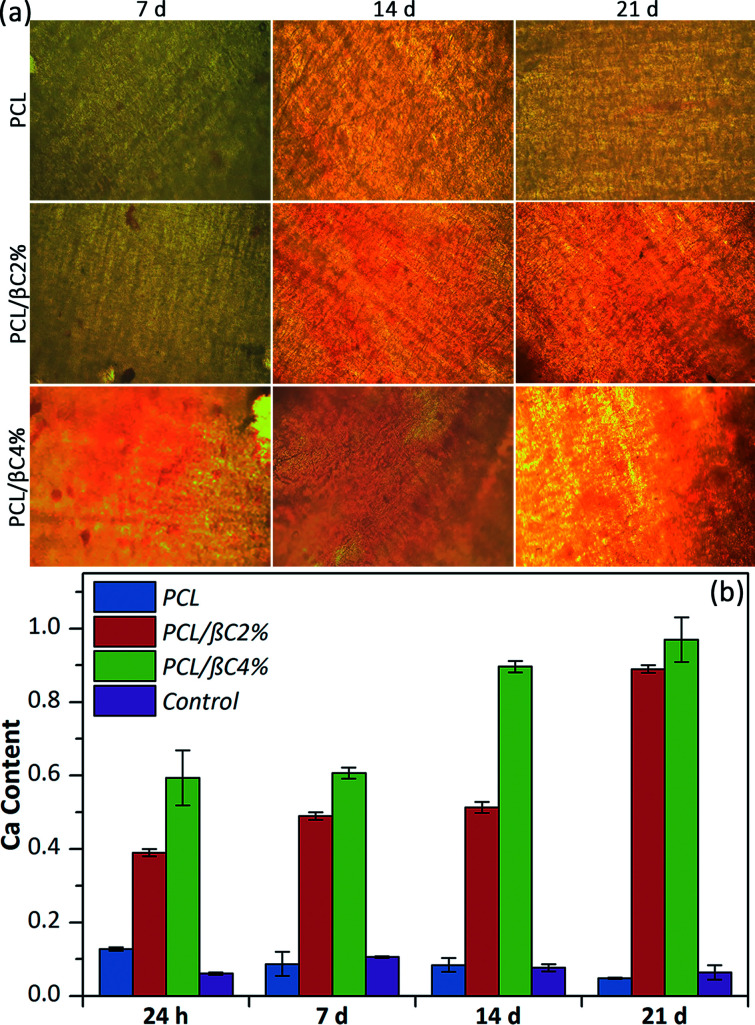
(a) Alizarin red staining of the scaffolds seeded with MSCs cells (10^4^ cells per well). The magnification is 200. (b) Calcium content of the scaffolds seeded with MSCs. Tissue culture plate was used as the control.

Alizarin red staining was performed to qualitatively evaluate the formation of mineralized matrix on the scaffolds. The results indicated the absorption of alizarin red dye on β-carotene containing scaffolds, particularly PCL/βC4%, due to the calcium deposed by osteodifferentiated cells (Fig. 4a). The intensity of absorbed dye increased over 21 days, indicating the progress of osteodifferentiation of cells during this period.

The quantity of calcium generated by the osteodifferentiated cells on scaffolds was measured *via* calcium content assay (Fig. 4b). The calcium content on scaffolds containing β-carotene increased over 21 days without using any external osteogenic differential agents, demonstrating the osteogenic osteoinductive effect of incorporated β-carotene for seeded MSCs. Moreover, low calcium content was detected for pure PCL scaffold, similar to the tissue culture plate, showing no differentiation of MSCs to osteoblasts.

**Fig. 5:**
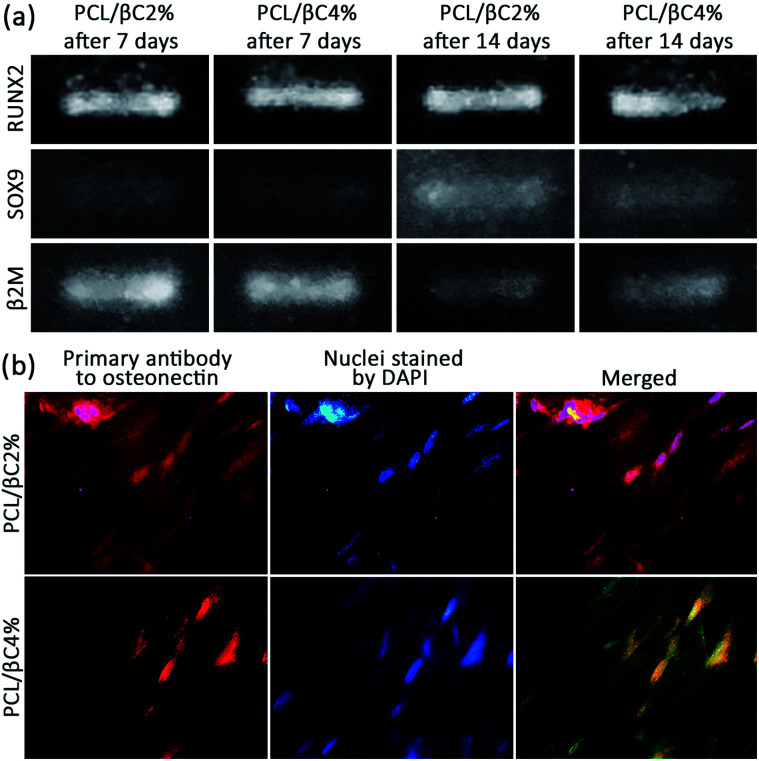
(a) RT-PCR results for expression of *RUNX2* and *SOX9* of osteodifferentiated cells (10^6^ cells per well) on scaffolds (0.5 × 0.5 cm^2^). The expression of β2M was recorded as a control gene. (b) ICC results for expression of *osteonectin* of osteodifferentiated cells on scaffolds after 21 days. The magnification is 400.

The osteoinductivity of β-carotene within scaffolds for MSCs was assessed by evaluating the expression of *RUNX2* and *SOX9* genes in cells through reverse transcription polymerase chain reaction (RT-PCR). To this end, the total RNA of the cells was isolated and converted into cDNA by reverse transcriptase enzyme and polymerase chain reaction was then performed on the cDNA. The expression of β2M was recorded as a control gene. *RUNX2* is the most important gene for following the osteogenic differentiation of MSCs.^42^ RT-PCR data (Fig. 5a) show the expression of *RUNX2* (band at 81 bp) for both PCL/βC2% and PCL/βC4% scaffolds after 7 and 14 days, confirming the osteoinductive effect of β-carotene within scaffolds on the differentiation of seeded MSCs. Nishide *et al.*^28^ also reported that β-carotene increased *RUNX2* expression in the MC3T3-E1 cell line. *SOX9* is another gene expressed only in the early phase of osteogenesis; thus, when the cells pass this phase, *SOX9* will no longer be expressed.^42^ RT-PCR data (Fig. 5a) display the expression of *SOX9* (band at 74 bp) for both PCL/βC2% and PCL/βC4% scaffolds even after 14 days, which indicates that the cells did not pass the early phase of osteogenesis and were still osteochondro-progenitor.

Finally, the expression of *osteonectin* gene, a differentiation marker for bone cells,^43^ was evaluated to approve the osteogenic differentiation of MSCs on PCL/βC2% and PCL/βC4% scaffolds. For this purpose, immunocytochemistry (ICC) assay was performed on cells seeded on scaffolds for 21 days. The fluorescence microscopic images (Fig. 5b) demonstrate the expression of *osteonectin* in a large number of cells, confirming the differentiation of seeded MSCs to osteoblasts as a result of the osteoinductive effect of incorporated β-carotene without any external differential agent.

It is worth mentioning that no degradation was observed in the PCL scaffolds during 21 days immersion in growth medium. Therefore, β-carotene should be released from the mats through a diffusion-out phenomenon before expressing its osteoinductive effect on the MSCs.

## Conclusion

The β-carotene-containing fibrous mats can be used as an excellent scaffold for bone tissue engineering to provide an appropriate environment for the osteodifferentiation of MSCs. The as-fabricated scaffolds were biocompatible and support the attachment and proliferation of seeded MSCs. Calcium content and ICC results confirmed the osteoinductive effect of β-carotene incorporated in the scaffolds on the differentiation of MSCs to osteoblasts without using any external osteogenic differential agent. However, RT-PCR results demonstrated that the cells did not pass the early phase of osteogenesis and were still osteochondro-progenitor after 21 days incubation.

## Conflicts of interest

There are no conflicts of interest to declare.

## Supplementary Material

RA-008-C7RA13237A-s001
