# Correction: β-Carotene: a natural osteogen to fabricate osteoinductive electrospun scaffolds

**DOI:** 10.1039/c8ra90032a

**Published:** 2018-04-25

**Authors:** Atiyeh Dabouian, Hadi Bakhshi, Shiva Irani, Mohamad Pezeshki-Modaress

**Affiliations:** Department of Biology, School of Basic Sciences, Science and Research Branch, Islamic Azad University 1477893855 Tehran Iran; Macromolecular Chemistry II, University of Bayreuth Universitätsstraße 30 95440 Bayreuth Germany hadi.bakhshi@uni-bayreuth.de; Burn Research Center, Iran University of Medical Science Tehran Iran

## Abstract

Correction for ‘β-Carotene: a natural osteogen to fabricate osteoinductive electrospun scaffolds’ by Atiyeh Dabouian *et al.*, *RSC Adv.*, 2018, **8**, 9941–9945.

The authors regret that the affiliation details for Mohamad Pezeshki-Modaress were incorrect in the original article. The correct author affiliations are as presented above.

The Royal Society of Chemistry apologises for these errors and any consequent inconvenience to authors and readers.

## Supplementary Material

