# Supplementary material for: β-Carotene: a natural osteogen to fabricate osteoinductive electrospun scaffolds
Source: RSC Adv. 2018 Mar 12;8(18):9941–5. doi: 10.1039/c7ra13237a (PMC9078714; doi:10.1039/c7ra13237a)
Supplement: RA-008-C7RA13237A-s001 [file RA-008-C7RA13237A-s001.pdf]

Electronic Supplementary Material (ESI) for RSC Advances.
This journal is © The Royal Society of Chemistry 2018

Supplementary Information

For

β-Carotene: a Natural Osteogen to Fabricate Osteoinductive Electrospun Scaffolds

Atiyeh Dabouian[1], Hadi Bakhshi*[,2], Shiva Irani*[,1], Mohamad Pezeshki-Modaress[3]

[1]Department of Biology, School of Basic Sciences, Science and Research Branch, Islamic Azad University, 1477893855 Tehran, Iran

[2]Macromolecular Chemistry II, University of Bayreuth, Universitätsstraße 30, 95440 Bayreuth, Germany

[3]Burn Research Center, Iran University of Medical Science, Tehran, Iran

*Corresponding authors: E-mail: (H. Bakhshi) hadi.bakhshi@uni-bayreuth.de, (S. Irani) s.irani@srbiau.ac.ir

22nd March 2018

Note added after first publication: This Supplementary Information file replaces that originally published on 12th March 2018, in which the author affiliation and author name for Mohamad Pezeshki-Modaress were incorrect. The correct details are as presented above.

1. Experimental

1.1. Materials

Polycaprolactone (PCL) with an average molecular weight of 80 kDa and β-carotene (βC, ≥97.0%) were obtained from Sigma-Aldrich. Human bone marrow-derived mesenchymal stem cells (hMSCs) were obtained from Royan Stem Cell Technology Company (Bon Yakhteh-e-Royan, Iran).

1.2. Fabrication of fibrous mats

Fibrous scaffolds were fabricated through electrospinning technique. For this purpose, homogeneous solutions of PCL (12.5 wt%) in chloroform/DMF mixture (2/1 v/v) containing different amount of β-carotene (0, 2 and 4 wt% regarding PCL) were prepared. Electrospinning was done at a voltage of 18 kV and a flow rate of 1 mL/h on a collector at distance of 20 cm rotating at 2000 rpm. The collected fibers were kept in darkness before use.

1.3. Characterization

Scanning electron microscopy (SEM) was carried out on a Tescan instrument (model Vega II, Czech). For cell imaging, the seeded scaffolds were washed with phosphate buffered saline (PBS, pH = 7.4) and the attached cells were fixed by glutaraldehyde solution (2.5%). Next, the fixed cells were dehydrated through serial dipping in ethanol solutions (60 to 100%) and dried overnight. The samples were coated with gold before imaging. SEM images were future analyzed by ImageJ software (version 1.51j8) to determine the fiber dimeter, pore size and porosity of mats.

1.4. Biocompatibility

The fabricated scaffolds (0.5×0.5 cm^2) were sterilized by immersing in ethanol 70%, deionized water, and PBS, respectively. MSCs were cultured in Dulbecco's modified eagle's medium (DMEM, Bioidea, Iran) containing 10% of fetal bovine serum (FBS, Gibco, Germany) in an incubator at 37°C under humidified CO_2 (5%) atmosphere. In the third passage, MSCs were seeded on the scaffolds in 96-well plates (10^4 cell/well) and maintained in the incubator up to 72 hours. The cell viability of cells was determined via MTT assay (3-[4,5-dimethylthiazol-2yl]-2,5-diphenyl tetrazolium bromide, Sigma-Aldrich) using an Elisa plate

reader (BioTek EL × 800) operating at wavelengths of 490/630 nm. Tissue culture plate was used as a control. The reported values are an average of three replicates.

1.5. Osteoblast differentiation

MSCs were seeded on scaffolds (0.5×0.5 cm^2) in 96-, 24- or 6-well plates (10^4 cell/well) and maintained in the incubator up to 21 days. To study the osteoinductive effect of incorporated β-carotene, DMEM medium with 10% of FBS was used as a non-differential medium and refreshed every 3 days. Meanwhile, for comparison, the osteogenic differentiation of MSCs was studied in a differential DMEM medium containing FBS (5%), L-ascorbic acid (10 mM, Sigma-Aldrich) and dexamethasone (1 Mm, Sigma-Aldrich) [1]. The viability of differentiated cells on scaffolds was determined via MTT assay. Tissue culture plate was used as a control. The reported values are an average of three replicates.

1.6. Alizarin Red staining

MSCs were seeded on scaffolds (0.5×0.5 cm^2) in 96-, 24- or 6-well plates (10^4 cell/well) and maintained in the incubator up to 21 days. The seeded scaffolds were washed with PBS, and the attached cells were fixed by paraformaldehyde solution (10%, Merck). The calcium cations generated by the differentiated cells were stained with Alizarin-Red (3,4-dihydroxy-9,10-dioxo-2-anthracenesulfonate sodium, Sigma-Aldrich) solution (10 g/L). The stained cells were observed under an inverted optical microscope (Motic, model AE31 Elite Trinocular, China). Tissue culture plate was used as a control.

1.7. Calcium content

MSCs were seeded on scaffolds (0.5×0.5 cm^2) in 96-, 24- or 6-well plates (10^4 cell/well) and maintained in the incubator up to 21 days. The seeded scaffolds were washed with PBS and shaken in HCl solution (0.6 M) at 4°C for 1-2 hours. The amount of calcium deposited on the scaffolds was measured via a calcium content kit (Pars Azmun, Iran) using an Elisa plate reader (BioTek EL × 800) operating at wavelengths of 570 nm. Tissue culture plate was used as a control. The reported values are an average of three replicates.

1.8. Reverse transcription polymerase chain reaction (RT-PCR)

MSCs were seeded on scaffolds (0.5×0.5 cm^2) in 6-well plates (10^6 cell/well) and maintained in the incubator up to 21 days. Total RNA of cells was isolated by an RNA extraction kit (Arya Tous, Iran) and converted to cDNA through reverse transcriptase enzyme (RT) using a cDNA synthesis Kit (Arya Tous, Iran). Polymerase chain reaction (PCR) was then performed on cDNA via a PCR master mix (Arya Tous, Iran). The PCR products were separated by agarose gel electrophoresis (Bio-Rad, USA), stained by SYBR green and photo-documented. Tissue culture plate was used as a control. The primers were designed by Gene Runner software (version 6.5.32), after obtaining the gene sequences from National Center for Biotechnology Information (NCBI) website (https://www.ncbi.nlm.nih.gov), and provided by Takapouzist (Iran): *RUNX2*, F: 5'-ATGGTTAATCTCCGCAGGTC-3', R: 3'-TACTGCACTGTGGTTAG-5', *SOX9*, F: 5'-GTACCCGCACTTGCACAACG-3', R: 3'-TCTCGCTCGTTCAGAAGTC-5', and *β2M* (as a control gene), F: 5'-TGCTGTCTCCATGTTTGATGTATCT-3', R: 3'-TCTCTGCTCCCCACCTCTAAGT-5'.

1.9. Immunocytochemistry (ICC)

MSCs were seeded on scaffolds (0.5×0.5 cm^2) in 24-well plates (10^4 cell/well) and maintained in the incubator up to 21 days. The seeded scaffolds were washed with PBS, and the attached cells were fixed by glutaraldehyde solution (2.5%). The scaffolds were then immersed in Triton solution (0.1 %) to increase the permeability of fixed cells and incubated first with an antibody against *Osteonectin* (Santa Cruz Biotechnology, USA) at 4°C overnight and then with secondary antibodies (conjugated with phycoerythrin, Chemicon International, USA) at 37°C for 4 hours. After washing with PBS and immersing in Triton solution (0.1 %), the nuclei of fixed cells were stained with DAPI (4,6-diamidino 2-phenylindole, Sigma-Aldrich, UK) solution in dark condition. The stained cells were kept in the dark and cold condition before imaging by a confocal fluorescence microscope (Nikon, Japan). Tissue culture plate was used as a control.

1.10. Statistical analysis

Statistical analyses were performed via PASW Statistics program package, version 18 (SPSS Inc., Chicago, IL, USA) using one-way ANOVA test. The significance level was set at P = 0.05 (95 % confidence intervals).

2. Results and Discussion

2.1. FTIR spectroscopy

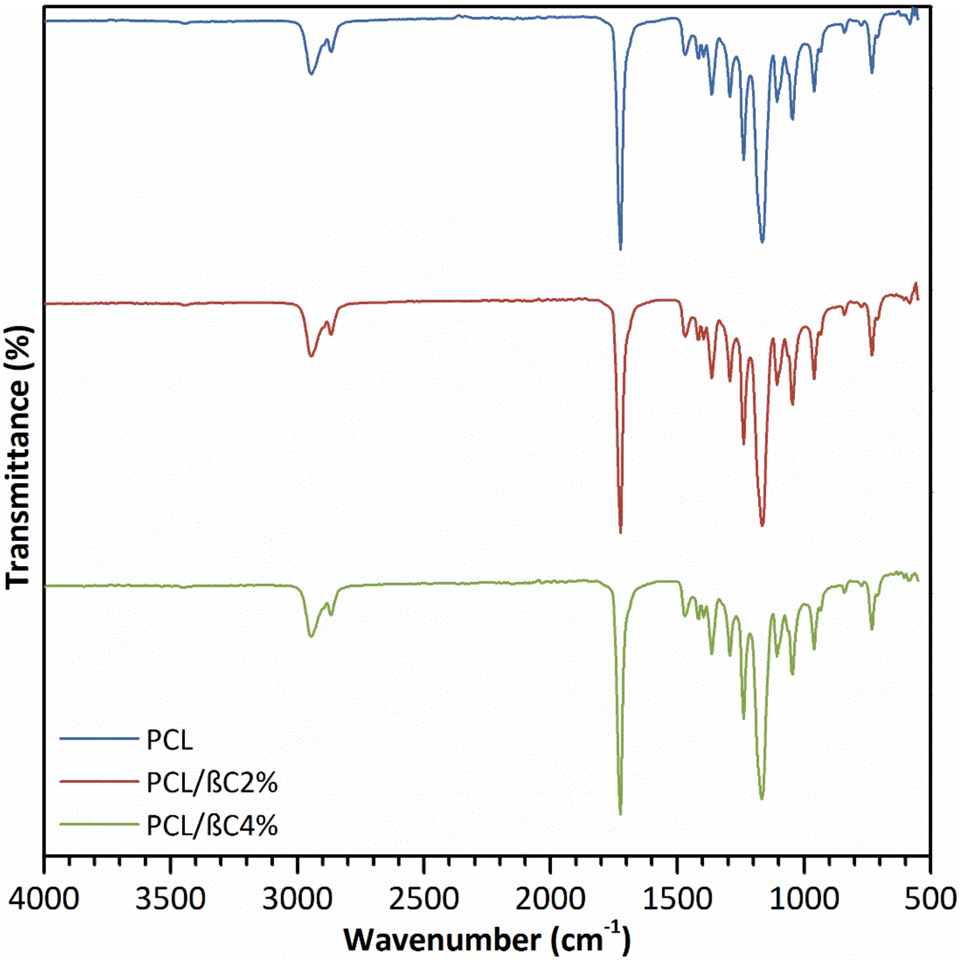

Figure S1. *FTIR spectra of PCL scaffolds.*

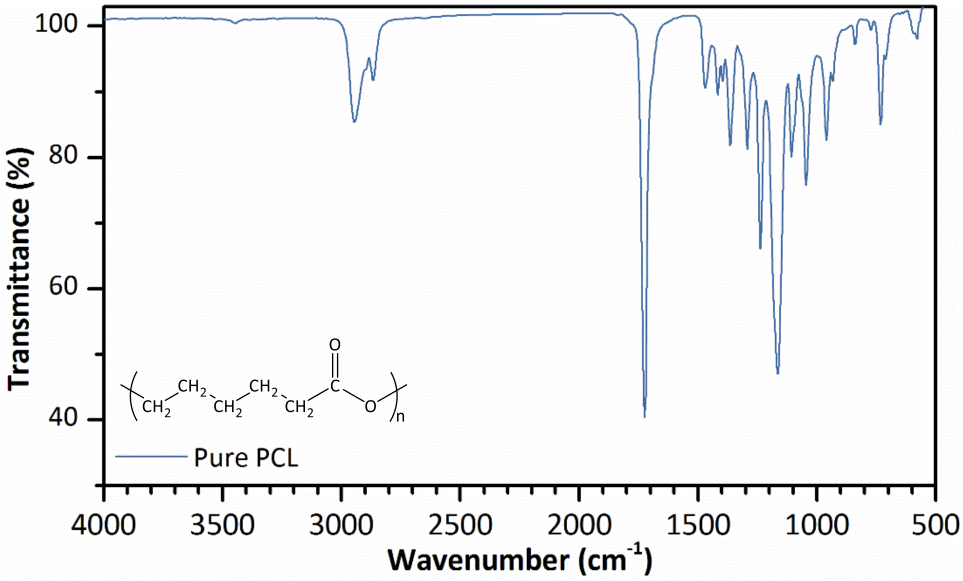

Figure S2. *FTIR spectrum of pure PCL and its chemical structure. 1/λ (cm^{-1}): 2943, 2867 (C–H, ν), 1724 (C=O, ν), 1470 and 1364 (CH_2, δ), 1165 (CO–O, ν) and 1046 (O–C, ν).*

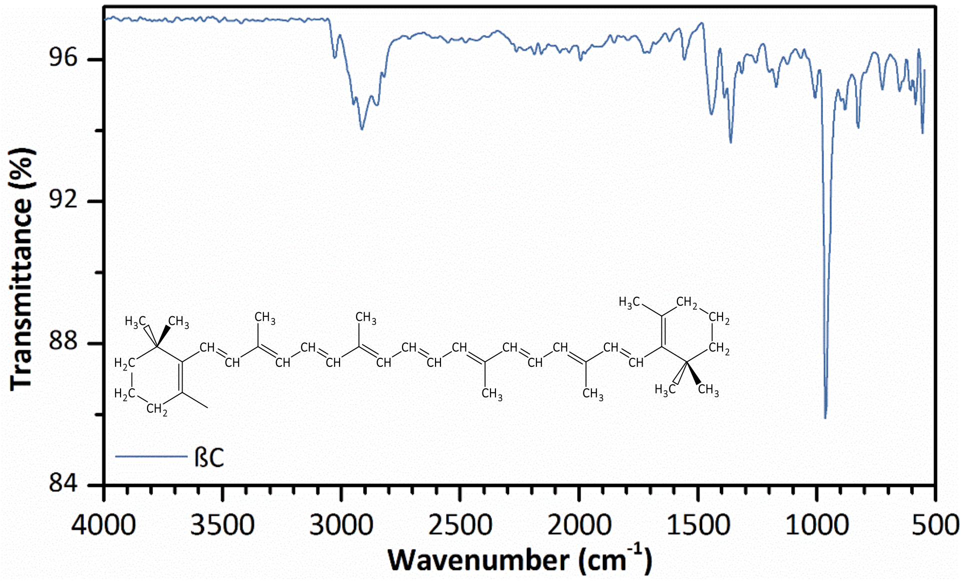

Figure S3. *FTIR spectrum of β-caroten and its chemical structure. 1/λ (cm^{-1}): 3028 (=C–H, ν), 2914, 2851 (C–H, ν), 1555 (C=C, ν), 1443 and 1362 (CH_2 and CH_3, δ) and 961 (=C–H, δ).*

3. References

1. B. Chuenjitkuntaworn, T. Osathanon, N. Nowwarote, P. Supaphol and P. Pavasant, *Journal of Biomedical Materials Research, Part A*, 2016 **104**, 264-271.
